# Assessing risk of vector transmission of Chagas disease through blood source analysis using LC-MS/MS for hemoglobin sequence identification

**DOI:** 10.1371/journal.pone.0262552

**Published:** 2022-01-24

**Authors:** Daniel Penados, José P. Pineda, Elisa Laparra-Ruiz, Manuel F. Galván, Anna M. Schmoker, Bryan A. Ballif, M. Carlota Monroy, Lori Stevens

**Affiliations:** 1 Faculty of Chemical Sciences and Pharmacy, Applied Entomology and Parasitology Laboratory, University of San Carlos, Guatemala City, Guatemala; 2 Faculty of Engineering, Science and Systems School, University of San Carlos, Guatemala City, Guatemala; 3 Department of Biology, University of Vermont, Burlington, Vermont, United States of America; Beni Suef University Faculty of Veterinary Medicine, EGYPT

## Abstract

Chagas disease is mainly transmitted by triatomine insect vectors that feed on vertebrate blood. The disease has complex domiciliary infestation patterns and parasite transmission dynamics, influenced by biological, ecological, and socioeconomic factors. In this context, feeding patterns have been used to understand vector movement and transmission risk. Recently, a new technique using Liquid chromatography tandem mass spectrometry (LC-MS/MS) targeting hemoglobin peptides has showed excellent results for understanding triatomines’ feeding patterns. The aim of this study was to further develop the automated computational analysis pipeline for peptide sequence taxonomic identification, enhancing the ability to analyze large datasets data. We then used the enhanced pipeline to evaluate the feeding patterns of *Triatoma dimidiata*, along with domiciliary infestation risk variables, such as unkempt piles of firewood or construction material, cracks in bajareque and adobe walls and intradomiciliary animals. Our new python scripts were able to detect blood meal sources in 100% of the bugs analyzed and identified nine different species of blood meal sources. Human, chicken, and dog were the main blood sources found in 78.7%, 50.4% and 44.8% of the bugs, respectively. In addition, 14% of the bugs feeding on chicken and 15% of those feeding on dogs were captured in houses with no evidence of those animals being present. This suggests a high mobility among ecotopes and houses. Two of the three main blood sources, dog and chicken, were significantly (*p < 0*.*05)* affected by domiciliary infestation risk variables, including cracks in walls, construction material and birds sleeping in the intradomicile. This suggests that these variables are important for maintaining reproducing *Triatoma dimidiata* populations and that it is critical to mitigate these variables in all the houses of a village for effective control of these mobile vectors.

## Introduction

Chagas disease is a debilitating, life-threatening, and neglected disease that affects vital organs, especially the heart and digestive tract. Chagas is caused by the parasite *Trypanosoma cruzi (Chagas*, *1909) (Kinetoplastida*, *Trypanosomatidae)* [1] and is endemic throughout Latin America, mostly in rural areas and especially in poor communities [2]. The drug treatments are mainly effective during the acute phase, with strong side effects in some cases [3]. The combined dynamics of the disease are estimated to cost 500 million US dollars annually across Latin America [4]. Currently the parasite can be transmitted by 154 species of blood feeding insect vectors of the subfamily Triatominae (Hemiptera: Reduviidae) [5]. Vector control is the most cost-effective strategy for disease prevention [2]; therefore, reducing the domiciliary infestation of Chagas vectors is a major objective [6].

*Triatoma dimidiata (Latreille*, *1811)* is the most important vector in Guatemala, and at least one million people live in *T*. *dimidiata* endemic regions [2]. The vector domiciliary infestation patterns and parasite transmission dynamics are complex and influenced by biological, ecological, and socioeconomic factors [7]. For effective disease control, the multitude of variables contributing to domiciliary infestation need to be addressed [7,8]. Long-term sustainable Chagas vector control in Central America is achieved by the Ecohealth approach, which tackles these biological, ecological, and socioeconomic factors, with community participation as a driving factor, integrating local communities and their customs [8–10] and acknowledging small scale differences in biological, ecological and socioeconomic variables [11] among locations and communities.

In particular, understanding vector peridomiciliary and intradomiciliary movement, colonization and blood meal sources are important for the success of vector control programs [8,12,13]. Various methods have been used to identify blood meal sources, including the Polymerase Chain Reaction (PCR) to target remnants of vertebrate DNA from traces of blood in the insect abdomen [8,12]. However, the detection ability of DNA decreases rapidly after feeding [13] and for some communities where blood meal sources may be scarce, over 60% of peridomicile and intradomicile collected bugs had no blood source detected using DNA based methods [8,11]. A new method for blood meal detection in Chagas vectors is now available, using liquid chromatography tandem mass spectrometry (LC-MS/MS) based on variation in vertebrate hemoglobin peptides. In a previous study, protein-based LC-MS/MS detected at least one blood source in 100% of the samples, and an average of 1.6 blood sources per bug, compared to 50% and 0.5 using DNA; however, the bioinformatic pipeline was not designed to analyze a large number of samples [13].

The aim of this study was to further develop the LC-MS/MS pipeline to analyze a greater number of samples. This updated pipeline was used to evaluate the feeding dynamics of *T*. *dimidiata* collected in a Chagas endemic village within the municipality of Comapa, Jutiapa, Guatemala, to understand vector intradomicile and peridomicile movement, colonization and Chagas parasite transmission risk.

## Methods

This study is part of a multidisciplinary, collaborative, inter-agency and comprehensive approach for the control of Chagas disease [14]. Specifically, this study focuses combining detection of blood meal sources with household surveys to understand how biological, ecological, and socioeconomic variables influence Chagas disease transmission dynamics. This study received ethical clearance from the San Carlos University bioethics committee (AC-010-2018), to work in Chagas endemic regions recommended by IPCAM-PAHO in 2014. Verbal consent was carried out, as requested from the San Carlos University bioethics committee, witnessed by the personnel of the Ministry of Health (MoH).

### Study area

Peridomestic and intradomestic areas were surveyed in the village of Anonito in the municipality of Comapa, department of Jutiapa, Guatemala (14,11871, -89,92522). Inhabited by mestizos (mixed race, Spanish and indigenous), they speak Spanish and are mainly engaged in agriculture. Jutiapa is a Chagas endemic region, with a largely rural population. As such, the villagers are predominantly poor, with 60% living in self-constructed houses built mainly of clay and vegetal materials such as “bajareque” and “adobe”. About 90% of these houses have notorious cracks in the walls, a major risk variable that promotes triatomine infestation and colonization [15].

Major economic activities in the region include subsistence farming with monocultures of corn (*Zea mays (Fl*., *1825*)), beans (*Phaseolus vulgaris*,*((L*. *1753))*, and “Jocote” (*Spondias purpurea*, *(L*., *1762))*, along with cattle breeding. All these activities have contributed to land use changes, including loss of forest coverage [16], which is now less than 25%, with the bugs being found almost exclusively in houses, raising the risk of parasite transmission to humans [17].

### Bugs collection and associated field data

Bugs were collected by three methods to maximize the number of houses where at least one bug was analyzed from each infested house. 1) Entomological survey: 86.6% of the houses in the village were systematically searched using the person-hour method by personnel from the vectors department of the MoH trained in safe handling of disease vectors using flashlights and forceps [18], in June 2018. We intended to survey the 100% of the houses in the village but the houses that were not surveyed were either uninhabited (11%) or Households that did not want to participate (2.4%). While the MoH personnel searched for bugs, a collaborator of the Laboratory of Applied Entomology and Parasitology (LENAP) surveyed a member of the household regarding Capacities, Aptitudes, and Practices (CAP) as previously described [7,11,17]. 2) Flush-out: After searching each house, the walls and roof were systematically sprayed with cypermethrin, an irritating insecticide, causing the bugs to emerge from their hiding places for capture [19,20], in November 2018. 3) Community participation: While doing the entomological surveys, personnel from MoH and LENAP provided collecting supplies and explained to residents how to collect and record relevant information for any bug found later in the house. These bugs were collected in a designated house in the village [21] and analyzed for this study if collected from houses without Entomological survey or Flush out bugs. Bugs included were collected between June 2018 and March 2019.

Upon collection, bugs were placed in mesh-covered plastic bottles labeled with the house identification number, name of the head of the household, date, and ecotope (intradomicile or peridomicile). The Entomological survey and Flush-out bugs were preserved individually the same day of collection in 95% ethanol, with capture and demographic information (sex or life stage) and whether it appeared engorged or not (indicating if it had fed recently) recorded in both a notebook and an electronic database. For Community participation bugs, days or weeks may have passed before being preserved in ethanol and recorded in the database.

The LC-MS/MS analysis was done for 1–3 randomly selected Entomological survey and Flush-out bugs from each infested house. The number of bugs analyzed for a house was based on the total number collected in that house, using Community participation bugs only when those were the only insects collected in that house.

### Entomological indexes

Four commonly used entomological indexes were calculated: Infestation = (Number of houses with bugs present/Total number of surveyed houses) x 100; Colonization = (Number of houses with nymphs present/Total number of infested houses) x 100; Intradomicile infestation = (Number of houses with bugs present inside the house/Total number of surveyed houses) x 100; Peridomicile Infestation = (Number of houses with bugs present in the peridomicile/Total number of surveyed houses) x 100.

### CAP survey

The CAP survey included socioeconomic variables, as well as Capacities, Aptitudes, and Practices related to Chagas disease and *T*. *dimidiata* infestation [17]. The analysis for this study focused on understanding the variation in vector blood meal sources and the presence of peridomicile and intradomicile animals, as these have been shown to influence house infestation [15]. The peridomicile and intradomicile animal presence was based on observations, answers provided by the house spokesperson and signs found inside the houses, such as animal nests or feces.

### Blood source identification

Bug blood sources were determined by identifying species specific vertebrate hemoglobin peptides using LC-MS/MS as described by [13,22] and summarized below.

#### Protein extraction, trypsin digestion and LC-MS/MS

Half of the last four segments of each insect abdomen were isolated using a razor blade and scissors which were cleaned after each sample with a 50% bleach solution. The insect tissue was mixed with 800 to 1200 μL of denaturing buffer (5% blue bromophenol, 150 mM Tris pH 6.8, 2% SDS, 5% β-mercaptoethanol, 7.8% glycerol) depending on the amount of tissue, vortexed for 20 seconds, and heated at 95° C for five minutes. The proteins were then separated via SDS-PAGE and stained with Coomassie blue. Hemoglobin (approximately 16 kDa) was excised from the gel for trypsin digestion, as previously described [13] The LC-MS/MS analysis was carried out as previously described [13], and employed a LTQ (linear trap quadrupole)-Orbitrap Discovery with a Finnigan Surveyor Pump Plus and Micro AS autosampler (Thermo Electron; San Jose, CA, USA) controlled with Xcalibur™ 2.1 Software (Thermo Fisher Scientific, Inc.; Waltham, MA, USA). Precursor ion spectra were obtained in the orbitrap, and fragment ion spectra were obtained in the linear ion trap.

#### Enhanced pipeline for peptide identification and determination of blood source taxa

Identification of the blood meal sources of a sample is possible because some tryptic hemoglobin peptides are conserved within species, but others vary between species, or even genus and family taxonomic divisions; thus, by evaluating the hemoglobin peptides in a sample we can identify the blood meal sources [13].

First, we used Proteome Discoverer 2.3 (Thermo Scientific) to search the raw data from each LC-MS/MS acquisition against a concatenated forward and reverse database of known hemoglobin sequences from GenBank [22] via SEQUEST. Dynamic modifications of methionine oxidation (+ 15.995 Da), and cysteine acrylamidation (+ 71.037 Da) and carbamidomethylation (+ 57.021 Da) were permitted, with no missed cleavages by trypsin. Precursor and fragment ion mass tolerances were set to ± 5 ppm and ± 0.02 Da, respectively. Lastly, high confidence cross-correlation score was used to filter the peptides.

In contrast to the previous analysis, which considered each sample individually [13] we collated the results into a multiconsensus report in Proteome Discoverer, comparing the presence or absence of each peptide across all samples, in effect streamlining our previous pipeline.

**Fig 1:**
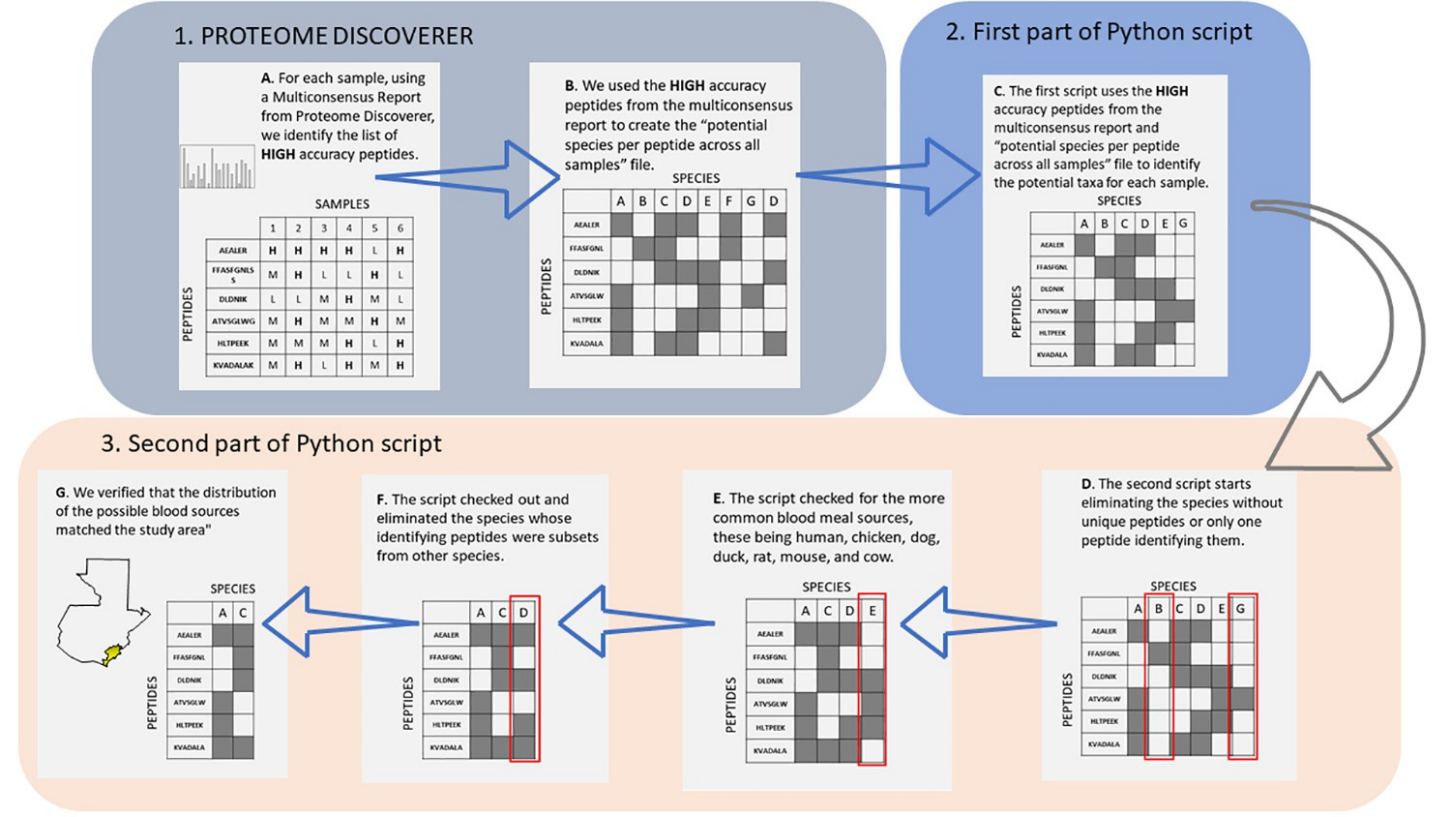
Graphic representation of the workflow for peptide taxonomic identification and principal blood sources identification.

**Fig 2:**
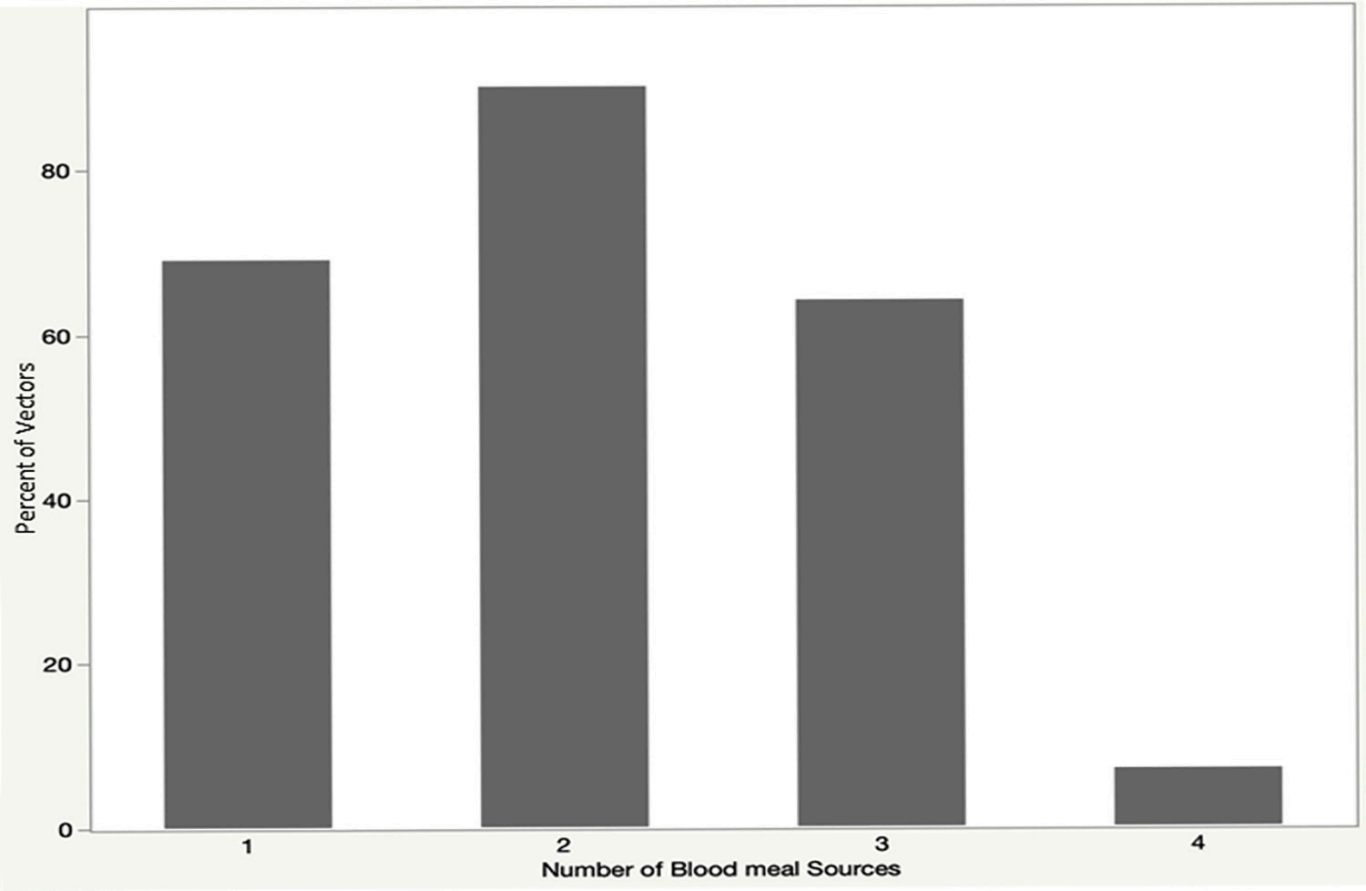
Percentage of bugs with one, two, three and four blood sources identified (N = 232).

Further enhancement of the pipeline included a novel python (v. 3.8.3 for Windows) script developed using the Pandas library (v. 1.1.5) to a) combine the multiconsensus report with peptide taxonomic maps and b) to identify the most likely blood meal sources. Previously, the process analyzed each sample individually, reducing the orders to major and minor blood meal sources. It then identified potential species for each blood meal from the reduced possible orders (see 1B in Fig 2, [13]). The novel pipeline uses two scripts. The first script loops through the samples in the multiconsensus report to match, by sample, the potential taxa for each peptide using an in-memory data structure (*pandas*.*DataFrame*). Because many of the peptides are common across multiple species, a second script was used to determine the most likely blood meal sources. First, it eliminated the species with only one peptide or no unique peptides. Second, the script checked for the more common blood meal sources, e.g. human, chicken, dog, duck, rat, mouse, and cow, based on our field observations during the surveys and our preliminary analysis [8,11]. Finally, because peptides can be misidentified by Proteome Discoverer and a species could have a previously unknown polymorphism, all potential blood meal sources were verified to have at least two unique peptides using nested loops. The remaining blood meal sources were considered valid unless the species geographic distribution did not include Jutiapa (Fig 1).

### Statistical analysis

Our analysis used subsets of the bugs focusing on different questions. To demonstrate the power of the enhanced pipeline, analyses of the entire data set (N = 232) included the average number of blood sources per bug and the different blood sources found in the vector population. To evaluate the relationship among blood sources we performed a multiple correlation analysis using the multivariate platform in JMP^®^ Pro (Version 15. SAS Institute Inc., Cary, NC, 1989–2021), using the probability value (P < 0.05) to assess the significance of the correlation between pairs of blood sources and the correlation value to determine if the relationship was negative or positive.

The remaining analyses focused on the Entomological survey bugs (N = 165), because we had information for the location where the bugs were collected (peri vs intradomicile) and the CAP survey data from the same house (e.g., presence of animals, and domiciliary infestations risk variables). To assess bugs’ movement, we compared the blood sources found in the bugs with the animals’ presence data obtained from the entomological survey, to identify if there were bugs feeding on animals absent from the house they were collected from, suggesting movement of the bug among houses.

Finally, to evaluate factors associated with colonization, that is, houses where the bugs are reproducing. The association of previously identified domiciliary infestation risk variables for *T*. *dimidiata* (adobe or bajareque walls, walls with cracks, firewood inside the house, construction materials inside the house, chicken inside the house, and dirt floor) [15,17] with the three main blood sources, *Homo sapiens* (L. 1758), *Gallus gallus* (L. 1758) and *Canis lupus* (L. 1758), was examined using logistic regression in R 3.6.3, with the function “glm” for a binomial distribution.

Statistical significance was determined by *p* < 0.05 except for the analysis related to colonization, because sample sizes were lower, we used *p* < 0.10 as the cutoff for significance.

## Results

Of the 209 houses in the village, 181 houses (86.6%) were surveyed, 23 were uninhabited and five were closed. Most (79.8%) of the bugs were found in cracks in rustic material walls (adobe or bajareque). We found that Anonito had a 36.5% infestation (houses with bugs present), 71.2% colonization (percentage of infested houses with nymphs), 36.5% intradomiciliary infestation and 1.66% peridomiciliary infestation.

The enhanced pipeline was able to identify at least one blood source in 100% of the samples (N = 232), even if they were not engorged (20.7%). The average number of blood sources per sample was 2.04. We identified only one blood source in 29.74%, two blood sources in 38.79%, three blood sources in 27.6% and four blood sources in 3.02% (Fig 2).

**Fig 3:**
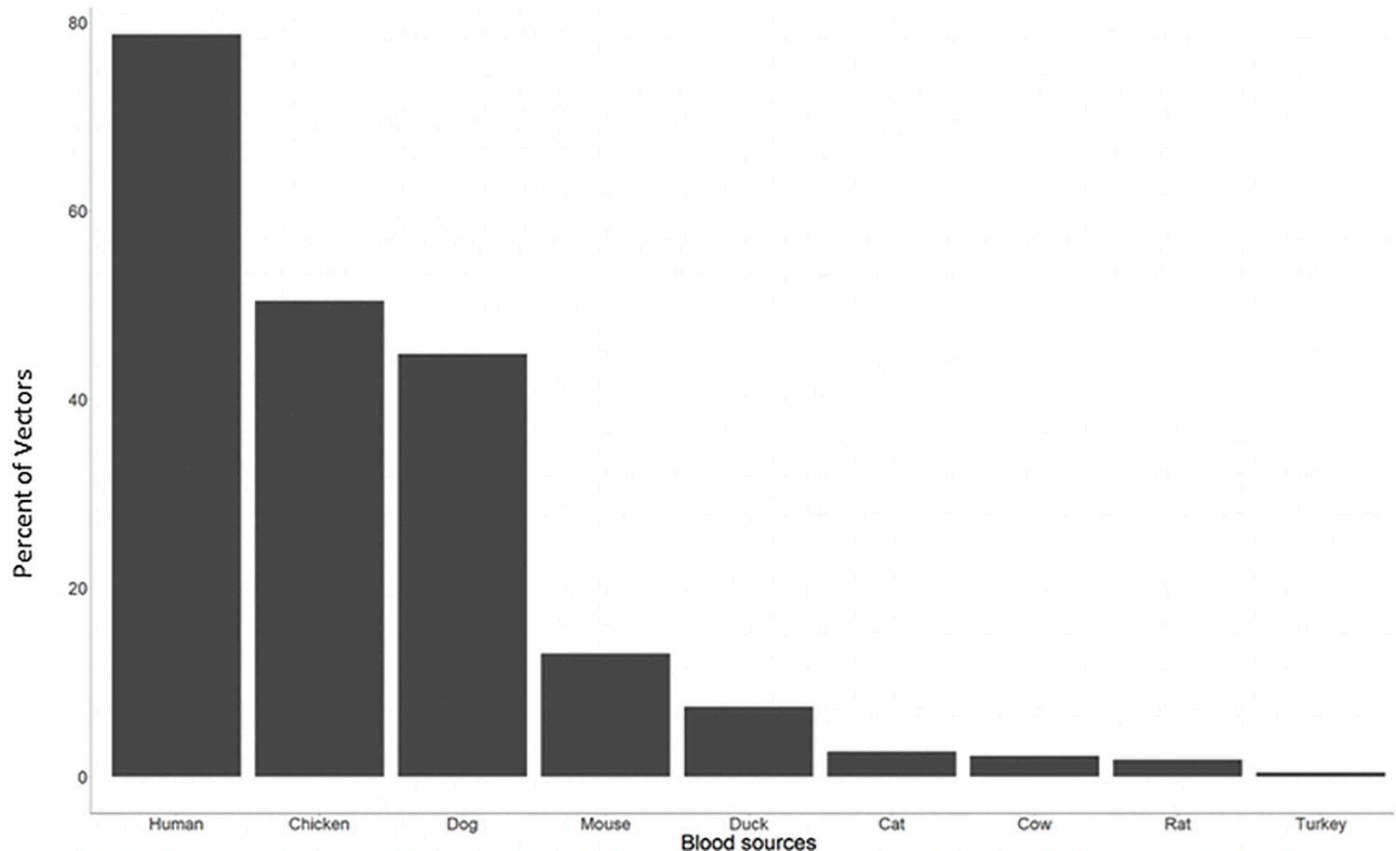
Percent of bugs with each blood source for bugs collected in Anonito, Jutiapa, Guatemala. Total percentage is over 100 because the average blood sources per bug was 2.06.

The enhanced pipeline was able to not only identify blood sources in 100% of the samples, but also identified nine different blood meal sources. The predominant blood source was *H*. *sapiens* (human), found in 78.7% of the insects, followed by chicken (*G*. *gallus)* found in 50.4% and dog (*C*. *lupus)* in 44.8%. Less common were *Mus musculus* (L. 1758) (white mouse) 13%, *Cairina moschata* (L. 1758) (duck) 7.4%, *Felis catus* (L. 1758) (cat) 2.6%, *Bos taurus* (L. 1758) (cow) 2.2%, *Rattus norvergicus* (Berkenhout, 1769) (rat) 1.7%, and *Meleagris gallopavo* (L. 1758) (turkey) 0.4% (Fig 3).

The correlation probability and the correlation value among the possible comparisons of the nine blood sources can be seen in the Table 1. Human and dog had a negative relationship (p<0.001), and human and mouse were positively related (p = 0.0032). Chicken and mouse were negative related (p = 0.0051), and chicken and duck were negative related (p = 0.0211). Lastly, duck and turkey were positively related (p<0.001).

**Table 1:** Multivariate correlations among blood sources (N = 232).

**Correlation Probability**									
	**human**	**dog**	**chicken**	**duck**	**cow**	**mouse**	**rat**	**cat**	**turkey**
human	**< .001**	**< .001**	0.9477	0.8021	0.0631	**0.0032**	0.8019	0.4102	0.6181
dog	**< .001**	**< .001**	0.0626	0.5327	0.5414	0.1476	0.4051	0.8294	0.3605
chicken	0.9477	0.0626	**< .001**	**0.0211**	0.9829	**0.0051**	0.9861	0.3981	0.3142
duck	0.8021	0.5327	**0.0211**	**< .001**	0.4853	0.5601	0.5707	0.3811	**0.0003**
cow	0.0631	0.5414	0.9829	0.4853	**< .001**	0.3386	0.7426	0.6862	0.8704
mouse	**0.0032**	0.1476	**0.0051**	0.5601	0.3386	**< .001**	0.4368	0.3386	0.6994
rat	0.8019	0.4051	0.9861	0.5707	0.7426	0.4368	**< .001**	0.7426	0.8945
cat	0.4102	0.8294	0.3981	0.3811	0.6862	0.3386	0.7426	**< .001**	0.8704
turkey	0.6181	0.3605	0.3142	**< .001**	0.8704	0.6994	0.8945	0.8704	**< .001**
**Correlation Value**									
human	**1.0000**	-0.3710	0.0043	0.0166	-0.1227	0.1936	-0.0166	-0.0546	0.0330
dog	-0.3710	**1.0000**	0.1230	0.0413	-0.0405	-0.0958	-0.0552	0.0143	-0.0606
chicken	0.0043	0.1230	**1.0000**	-0.1520	-0.0014	-0.1841	-0.0012	-0.0560	-0.0667
duck	0.0166	0.0413	-0.1520	**1.0000**	-0.0462	0.0386	-0.0376	0.0580	0.2339
cow	-0.1227	-0.0405	-0.0014	-0.0462	**1.0000**	-0.0634	-0.0218	-0.0268	-0.0108
mouse	0.1936	-0.0958	-0.1841	0.0386	-0.0634	**1.0000**	-0.0515	-0.0634	-0.0256
rat	-0.0166	-0.0552	-0.0012	-0.0376	-0.0218	-0.0515	**1.0000**	-0.0218	-0.0088
cat	-0.0546	0.0143	-0.0560	0.0580	-0.0268	-0.0634	-0.0218	**1.0000**	-0.0108
turkey	0.0330	-0.0606	-0.0667	0.2339	-0.0108	-0.0256	-0.0088	-0.0108	**1.0000**

The correlations are estimated by Row-wise method.

All the bugs with *cow* as a blood source were collected in houses that did not have cows present either inside or outside of the house, and 33.3% of these bugs were nymphs (Table 2). On the same note, the bugs that had *dog* reported as a blood source were captured in a total of 65 different houses, with 12 (18.46%) of these houses having no dogs reported on their surroundings, and 64.7% of these bugs were nymphs. In the case of *chicken*, the bugs that were feeding from this species were captured in 58 different houses, with 14 (24.13%) of them having no presence of chickens reported, and 65.2% of these bugs being nymphs. The bugs feeding on *cat* were captured on six different houses, but two of these (33.33%) did not have cats reported, and 50% of these bugs were nymphs.

**Table 2:** Presence of species compared to the blood sources identified.

Blood meal source	Houses of origin Houses with bugs that had fed on blood meal source	Houses without the blood meal species	Percentage	Nymphs percentage
Cow	6	6	100%	33.33%
Cat	6	2	33.33%	50.00%
Dog	65	12	18.46%	64.70%
Chicken	58	14	24.13%	63.15%

The factors associated with colonization identified by logistic regression evaluating the relationship of the domiciliary risk variables with the main blood sources are shown in Table 3. Cracks in walls had an association with bugs feeding on chicken, while birds (chickens and/or ducks) and construction materials in the intradomicile had a significant association with feeding on dog, and construction material in the intradomicile had a significant association with feeding on human.

**Table 3:** Logistic regression results evaluating the association of the domiciliary risk variables with the main blood sources (human, chicken, dog) (N = 90).

Independent Variable	Dependent Variable	*p value*
Domiciliary risk Variable	Blood Source	
Rustic material walls like *Adobe* or *Bajareque*	Human	0.144
	Chicken	0.45
	Dog	0.447
Cracks in walls	Human	0.846
	Chicken	0.001***
	Dog	0.314
Firewood in the intradomicile	Human	0.217
	Chicken	0.464
	Dog	0.412
Construction material in the intradomicile	Human	0.092
	Chicken	0.911
	Dog	0.032**
Chicken in the intradomicile	Human	0.086*
	Chicken	0.174
	Dog	0.004***
Dirt floor	Human	0.456
	Chicken	0.213
	Dog	0.823

Significance codes <0.1 ’*’; < 0.05 ’**’; <0.01 ’***’.

## Discussion

The enhanced pipeline was able to identify blood sources in 100% of the analyzed bugs, including bugs that had no evidence of recent feeding (not engorged) and or bugs that were not preserved in ethanol for several days. It identified nine species, an average of 2.06 blood meal sources per bug and two or more blood sources in more than 70% of the bugs. This contrasts with DNA analysis of previous studies, where blood sources were only found in 60% of bugs and only one blood source was identified in most [11,13]. This result reinforces the power of the mass spectrometry analysis to understand the feeding dynamics of triatomines. Moreover, the newly developed python script makes the technique practical for large epidemiological studies necessary to understand *T*. *dimidiata* dynamics in the region.

The entomological survey data indicated that Anonito is more highly infested in comparison with previously studied villages from the same region [23]. The high colonization index (>70% of infested houses had nymphs as evidence of reproduction within the house) indicates a population highly adapted to houses. Further supporting adaptation to houses is the higher intradomiciliary infestation (36.5%) compared to peridomiciliary infestation (1.66%), which is a pattern previously reported [17,24]. The most important intradomiciliary risk variable are cracks in *abode* or *bajareque* walls, where more than 80% of the bugs were found, a pattern found in previous studies [15].

We found evidence of nine different blood sources in just one village, indicating that *T*. *dimidiata* is an opportunistic feeder, where its high mobility helps finding different blood sources [23]. In addition, despite that ~80% of the bugs were found in the intradomicile, the variety of peridomestic and intradomestic blood sources in these bugs inside houses indicates its moving capacity, which means that the vector control mechanisms should be done in both intra and peri domestic ecotopes together [11]. The three main blood sources of the bugs from Anonito were human, chicken and dog, found in 78.7%, 50.4% and 44.8%, respectively. This is the same pattern found in many other studies of *T*. *dimidiata*, using different techniques [11,13,25], reinforcing the importance of these three species in the behavior and vector dynamics of domiciled *T*. *dimidiata*.

The negative relationship between human and dog blood sources (Table 1), indicates that when bugs are feeding on dogs, they do not feed on humans. Also, chicken and mouse were negatively related, like chicken and duck. These could be indicating that a bug does not need several small blood feedings if one blood source provides a complete meal. The first species that the bug detects after awakening is the most likely to function as a complete meal [26]. This could mean that eliminating hiding places and animals from the intradomicile will facilitate that the bug detects domestic animals in the peridomicile first, reducing human as the principal blood source [8,26]. This is just a hypothesis, and the more sensitive analysis of LC-MS/MS for hemoglobin sequence identification will be very valuable for analyzing these questions.

The absence of non-domiciled blood sources such as opossum and bats [27] or any common wild mouse in the region, such as *Peromyscus* spp., previously reported as blood sources, is notable. We observed that only 2% of the triatomines fed from cows, as seen in Fig 2. In this region, the cattle pastures are located far away from the villages and cattle are not found in villages [17]. This means that there is some evidence of bugs moving from far away from the village to inside of the village. This matches with Cahan et al (2019) and Penados et al (2020), where they describe that there is no strong evidence, in Jutiapa, that *T*. *dimidiata* comes from outside the village [17,28].

The evidence of a bug found in the intradomicile that had fed from human (strictly intradomiciliary blood source) and cow (strictly, peridomiciliary blood source) indicates that the bug moved between different ecotopes. We observed that 18% of the triatomines that fed from dogs were found in houses with no dogs in them, 24% for chickens, 33% for cats, and 100% for cows (Table 2). Although cats and dogs may roam freely among houses, it is not likely that chicken or cows move among houses. This suggests high mobility between houses and between ecotopes. This knowledge is crucial for vector control, because it means that individual interventions for specific houses, such as selective insecticide application, are not sufficient for eliminating Chagas transmission, because bugs can move from nearby infested houses [6]. Interventions that manage domiciliary risk variables in all the houses of a village will sustain the elimination of vector transmission risk in a village [29]. Moreover, more than 50% of the bugs that fed from animals that were not present in the house where they were collected were nymphs. This evidence shows that the nymphs move as much as adults. Further study could distinguish if this is an active or passive movement (e.g., the bugs are moved in firewood or other objects carried between houses).

Dog and human blood meals are related to accumulated construction material, suggesting that such materials provide a hiding place for bugs that feed on dog and human. Finally, chicken blood meals were related to cracks in walls, supporting the hypothesis that cracks are a hiding place for bugs that feed on chicken and eliminating animals from inside of the house and reducing the presence of risk variables could reduce the intradomiciliary infestation and the frequency of humans as a blood source. Therefore, reducing the vectorial transmission risk of Chagas disease [8].

Education and community participation are core components of our Ecohealth program that aims to eliminate risk factors as a key strategy in reducing vectorial transmission of Chagas disease in Guatemala [30]. The understanding of vector feeding patterns has been important for comprehending domiciled vector dynamics, and evaluating which factors to target to eliminate Chagas vectorial transmission in a community [8,11]. The use of mass spectrometry of hemoglobin was already demonstrated as an improvement over DNA based methods for blood sources identification in vectors [13]; however, in order to apply that technique to a high infestation village like Anonito (232 bugs analyzed), the taxonomic identification pipeline needed to be automated. The enhanced pipeline made possible by the new python script did that, enabling mass spectrometry to be used with large sample numbers in Chagas epidemiology studies.

The blood sources analysis reinforces multiple previous studies. Cracks in walls, intradomiciliary animals, and construction material in the intradomicile represent a risk for maintaining vector populations which are highly adapted to domiciles [15,17]. Eliminating these risk variables from the houses, with community participation as a key strategy, is imperative to reduce the transmission risk, evidenced by the high percentage of bugs feeding from humans in the village.
